# Assessment of 5-year outcomes of life satisfaction in survivors after rehabilitation programs: a multicenter clinical trial

**DOI:** 10.1038/s41598-022-05355-z

**Published:** 2022-01-27

**Authors:** Farshid Rahimi-Bashar, Mahmood Salesi, Keivan Gohari-Moghadam, Ali Fathi Jouzdani, Mohamad Amin Pourhoseingholi, Amir Vahedian-Azimi

**Affiliations:** 1 Department of Anesthesiology and Critical Care, School of Medicine, Hamadan University of Medical Sciences, Hamadan, Iran; 2 Chemical Injuries Research Center, Life Style Institute, Baqiyatallah University of Medical Sciences, Tehran, Iran; 3 Medical ICU and Pulmonary Unit, Shariati Hospital, Tehran University of Medical Sciences, Tehran, Iran; 4 Student Research Committee, Hamadan University of Medical Sciences, Hamadan, Iran; 5 Gastroenterology and Liver Diseases Research Center, Research Institute for Gastroenterology and Liver Diseases, Shahid Beheshti University of Medical Sciences, Tehran, Iran; 6 Trauma Research Center, Nursing Faculty, Baqiyatallah University of Medical Sciences, Sheykh Bahayi Street, Vanak Square, P.O. Box 19575-174, Tehran, Iran

**Keywords:** Diseases, Health care, Medical research

## Abstract

Using a rehabilitation program for the survivors of acute respiratory distress syndrome (ARDS) could be one of the important and fundamental steps to improve the pulmonary function and health-related quality of life (HRQoL) of patients. This study was carried out to evaluate the effect of two rehabilitation techniques (Family-Based Empowerment Model (FECM)/Continuing Care Model (CCM), or both of them) on pulmonary function, and HRQoL in ARDS survivors. From December 2009 to June 2016, ARDS survivors from mixed medical-surgical ICUs at four academic teaching hospitals in Tehran, Iran, were randomly assigned to one of three intervention groups (A, B, or C) or a control group (D). Pre- and post-interventions, pulmonary functions and HRQoL status of patients in all groups were collected 48 times via clinical measurements and various questionnaires during 5 years of follow-up. Significantly improvement was seen in the intervention groups compared to the control group, and the greatest benefit was observed in patients who received mixed of FCEM and CCM rehabilitation techniques. Co-administration of FCEM and CCM can improve pulmonary function as well as the life satisfaction of ARDS survivors. As a result, the execution of the empowerment model by nurses is recommended for ARDS survivors and the participation of their families at the same time.

**Trial registration:** NCT02787720 (ClinicalTrial.gov, 24/05/2016).

## Introduction

Acute respiratory distress syndrome (ARDS), as it is currently defined by Berlin criteria^1^, is characterized by: (a) *Timing* within 1 week of known clinical insult or new or worsening symptoms; (b) *Chest imaging* with bilateral opacities not fully explained by effusions, lobar/lung collapse, or nodules; (c) *Origin of edema* not fully explained by cardiac failure or fluid overload; and Oxygen impairment defined as mild (200 mmHg < partial pressure of alveolar oxygen (PAO_2_)/fraction of inspired oxygen (FiO_2_) ≤ 300 mmHg, with positive end-expiratory pressure (PEEP) or continuous positive airway pressure (CPAP) ≥ 5 cmH_2_O), moderate (100 mmHg < P/F ≤ 200 mmHg, with PEEP ≥ 5 cmH_2_O), or severe(PaO2/FiO2 ≤ 100 mmHg, with PEEP ≥ 5 cmH_2_O)^2^. It may occur in the setting of varied physiologic insults including pneumonia, sepsis, trauma or massive transfusion, and survivors often face a prolonged recovery course which includes reduced health-related quality-of-life (HRQoL), including disturbances to physical, mental (anxiety, depression, posttraumatic stress disorder [PTSD]) and social health^3–7^. Moreover, pulmonary function impairment and reduced exercise capacity had been found in about half of the survivors of ARDS^8^. At 1-year following hospital discharge, the patients recovering from ARDS have demonstrated evidence on pulmonary function testing (PFT) of reduced diffusing capacity of carbon monoxide (DLCO; up to 80%), airflow obstruction (up to 20%), and chest restriction (up to 20%)^9,10^. As such, rehabilitation practices have focused on both improving pulmonary function, and HRQoL, an increasingly important and useful measure of pulmonary rehabilitation that complements traditional ‘hard outcomes’ (e.g., mortality) to evaluate the impact of disease and benefits of medical interventions^11,12^.

Two contemporary models of the rehabilitation include the continuous care model (CCM)^13^, and the family-centered empowerment model (FECM)^14^. CCM model focuses on the effective, interactive, and balanced roles of healthcare providers, the patient, and the support structure so as to effectively influence the patients’ attitude toward the disease, improve treatment compliance and participation. This model was associated with improved HRQoL in renal transplant recipients^15^, patients following coronary artery bypass graft surgery^16^, and the patients with diabetes mellitus^17^.

FCEM was developed to improve the care and outcomes of the patients with chronic diseases and has previously been evaluated and validated in several chronic disease states^14^. Family engagement in the rehabilitation process may potentially have beneficial effects, including the improvements in mental and physical function, and reduced burden of disease^18,19^. The primary aim of model is to empower the patient/family unit to promote health quality. The model has four stages: (a) determining perceived threat (group discussion method); (b) self-efficacy (problem-solving method); (c) improving self-esteem (educational participation method) and (d) process and outcome evaluations^14^.

Little data is available comparing the effectiveness of pulmonary rehabilitation models in the ARDS survivors. We investigated the impact of pulmonary rehabilitation programs on ARDS survivors using either FCEM and CCM models or a hybrid FCEM-CCM model as compared to routine care on HRQoL.

## Results

### Participants of the study

**Figure 1:**
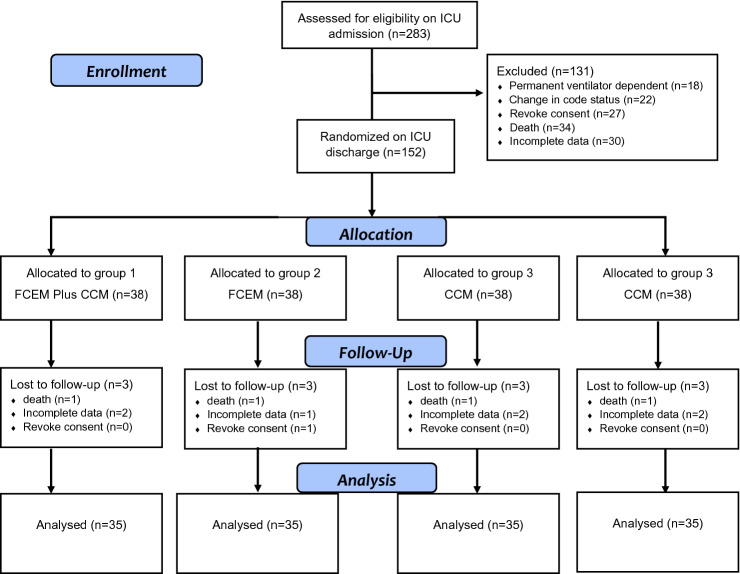
The flowchart of participants in the trial study.

From December 2009 to June 2016, 283 out of 367 ARDS survivors in the mixed medical-surgical ICU at four academic teaching hospitals in Tehran, Iran, met the inclusion criteria. Two-hundred eighty-three eligible patients were included and evenly randomized into four groups. A total of 143 patients due to reasons such as permanent ventilator dependence (n = 21), change of code status from "full code" (n = 27), revoked consent (n = 23), death (n = 24), and incomplete data (n = 48), were excluded from the final analysis. Therefore, 35 patients remained in each group, and 140 patients entered the final analysis. Figure 1 shows the flowchart of participants in the trial study.

### Demographic and clinical characteristics at baseline and over 5-years

140 subjects were included in the analysis. The mean age of the participants was 62 ± 8 years, with more than half of the patients was female (n = 82, 58.1%). Demographic and clinical characteristics at the baseline are presented in Table 1 for all groups of study. Patient’s demographics were similar among groups, including age (*P* = 0.165), sex (P = 0.525), BMI (*P* = 0.590), pre-ARDS BMI ≥ 30 (*P* = 0.24), ARDS risk factors (*P* = 0.85), family size (*P* = 0.12), marriage status (*P* = 0.156), urban residence (*P* = 0.63), and working full-time or part-time (*P* > 0.05). In addition, no significant difference was observed among the groups of study in terms of clinical characteristics. The only exception was in renal replacement therapy, which was significantly different among the groups (*P* = 0.043).

**Table 1:** Demographic and clinical characteristics at the baseline for all groups of study.

Variables	Group A	Group B	Group C	Group D	Total	P-value
Gender, female (%)	24 (68.6)	20 (57.1)	20 (57.1)	18 (51.4)	82 (58.6)	0.525
Marriage, married (%)	14 (40)	17 (48.6)	21 (60)	23 (65.7)	75 (53.6)	0.163
Living, City (%)	19 (54.3)	16 (45.7)	14 (40)	18 (51.4)	67 (47.9)	0.639
BMI ≥ 30 before ARDS, No (%)	21 (60)	27 (77.1)	28 (80)	26 (74.3)	102 (72.9)	0.242
**Cause of ARDS** (%)						
Pneumonia	8 (22.9)	2 (5.7)	3 (8.6)	4 (11.4)	17 (12.1)	0.859
Sepsis	10 (28.6)	9 (25.7)	8 (22.9)	9 (25.7)	36 (25.7)	
Trauma or burn	8 (22.9)	13 (37.1)	11 (31.4)	11 (31.4)	43 (30.7)	
Pancreatitis	7 (20)	8 (22.9)	10 (28.6)	9 (25.7)	34 (24.3)	
Others	2 (5.7)	3 (8.6)	3 (8.6)	2 (5.7)	10 (7.1)	
Any renal replacement therapy, yes (%)	23 (65.7)	26 (74.3)	19 (54.3)	15 (42.9)	83 (59.3)	0.043
Any paralytic agent, yes (%)	21 (60)	22 (62.9)	20 (57.1)	20 (57.1)	83 (59.3)	0.995
Any systemic glucocorticoid therapy, no (%)	18 (51.4)	21 (60)	18 (51.4)	21 (60)	78 (55.7)	0.791
Tracheostomy, no (%)	19 (54.3)	17 (48.6)	14 (40)	18 (51.4)	68 (48.6)	0.659
HFO technique, no (%)	20 (57.1)	19 (54.3)	18 (51.4)	16 (45.7)	73 (52.1)	0.801
Working full-time before ARDS, yes (%)	19 (54.3)	25 (71.4)	14 (40)	20 (57.1)	78 (55.7)	0.070
Working part-time before ARDS, no (%)	24 (68.6)	28 (80)	19 (54.3)	21 (60)	92 (65.7)	0.124
Age, mean (SD)	62.89 (10.16)	61.66 (6.89)	59.29 (6.45)	62.94 (6.75)	61.69 (7.77)	0.165
Family number, mean (SD)	5.2 (1.94)	5.51 (1.8)	5.8 (1.39)	6.11 (1.37)	5.66 (1.66)	0.120
BMI, mean (SD)	23.97 (3.2)	23.37 (2.05)	23.65 (3.46)	23.14 (1.41)	23.53 (2.65)	0.590
ICU length of stay, mean (SD)	56.43 (6.97)	54.8 (6.4)	55.57 (3.53)	55.8 (7.45)	55.65 (6.23)	0.752
Non-ICU length of stay, mean (SD)	20.71 (5.11)	19.4 (4.21)	21.94 (14.64)	22.97 (12.24)	21.26 (10.08)	0.486
SAPS III first days, mean (SD)	31.34 (4.26)	32.8 (8.24)	31.8 (6.7)	34.14 (8.91)	32.52 (7.25)	0.384
SOFA II first days, mean (SD)	15.91 (3.57)	14.8 (1.57)	14.71 (3.63)	15 (3.96)	15.11 (3.32)	0.411
APACHE IV first days, mean (SD)	24.54 (2)	25.14 (2.47)	24.49 (2.21)	24.57 (1.75)	24.69 (2.12)	0.537
LIS, mean (SD)	3.06 (0.64)	3.03 (0.62)	3.03 (0.57)	2.91 (0.66)	3.01 (0.62)	0.780
MODS, mean (SD)	14.06 (1.39)	14.03 (0.99)	13.78 (0.94)	14.49 (1.4)	14.09 (1.21)	0.097

Group A = FCEM + CCM; Group B = FCEM; Group C = CCM; Group D = routine care.

HFO: High frequency oxygenation; BMI: Body mass index; ICU: Intensive care unit; SAPS: Simplified Acute Physiology Score; SOFA: Sequential Organ Failure Assessment; APACHE: Acute Physiology and Chronic Health Evaluation; LIS: Lung Injury Score; MODS: Multi Organ Dysfunction Score.

Table 2 are shown the results of repetition a 5-year summary of demographic and clinical variables, including organ dysfunction, pulmonary dysfunction, coexisting illness, smoking status, and ability to return to work (Generalized Estimation Equations (GEE) model and Panel analysis are available in Supplementary Table 2). All 3 groups as intervention groups (groups A, B and C) were merged and compared to the control group (group D) in both models as the additional analysis. Return to work differed significantly between intervention and control groups, with both GEE and Panel analysis showing a higher chance of returning to work for group A than groups B, C, and D. The likelihood of returning to original work was greatest for Group A, followed by groups B, C, and D. The results held true for the groups A, B, and C combined vs. group D as controls.

**Table 2:** Changes in demographic and clinical characteristics over 5-year follow-up based on rehabilitation models in four groups of study.

Variables	Years		Group A	Group B	Group C	Group D	P-value*
Returned to work	First, yes (%)		27 (77.1)	14 (40)	18 (51.4)	0 (0)	< 0.0001
	Second, yes (%)		30 (85.7)	23 (65.7)	24 (68.6)	3 (8.6)	< 0.0001
	Third, yes (%)		32 (91.4)	26 (74.3)	28 (80)	5 (14.3)	< 0.0001
	Fourth, yes (%)		33 (94.3)	30 (85.7)	33 (94.3)	7 (20)	< 0.0001
	Fifth, yes (%)		33 (94.3)	32 (91.4)	34 (97.1)	11 (31.4)	< 0.0001
Returned to original work	First, yes (%)		8 (22.9)	7 (20)	11 (31.4)	0 (0)	0.006
	Second, yes (%)		19 (54.3)	13 (37.1)	16 (45.7)	0 (0)	< 0.0001
	Third, yes (%)		22 (62.9)	18 (51.4)	19 (54.3)	0 (0)	< 0.0001
	Fourth, yes (%)		27 (77.1)	23 (65.7)	26 (74.3)	1 (2.9)	< 0.0001
	Fifth, yes (%)		31 (88.6)	25 (71.4)	29 (82.9)	5 (14.3)	< 0.0001
Coexisting illness	First	No (%)	9 (25.7)	6 (17.1)	9 (25.7)	10 (28.6)	0.963
		One (%)	6 (17.1)	7 (20)	7 (20)	6 (17.1)	
		Two (%)	11 (31.4)	16 (45.7)	12 (34.3)	12 (34.3)	
		≥ Two (%)	9 (25.7)	6 (17.1)	7 (20)	7 (20)	
	Second	No (%)	12 (34.3)	7 (20)	9 (25.7)	4 (11.4)	0.001
		One (%)	13 (37.1)	14 (40)	17 (48.6)	13 (37.1)	
		Two (%)	10 (28.6)	14 (40)	9 (25.7)	11 (31.4)	
		≥ Two (%)	0 (0)	0 (0)	0 (0)	7 (20)	
	Third	No (%)	14 (40)	9 (25.7)	12 (34.3)	1 (2.9)	< 0.0001
		One (%)	15 (42.9)	15 (42.9)	17 (48.6)	13 (37.1)	
		Two (%)	6 (17.1)	11 (31.4)	6 (17.1)	15 (42.9)	
		≥ Two (%)	0 (0)	0 (0)	0 (0)	6 (17.1)	
	Fourth	No (%)	18 (51.4)	10 (28.6)	13 (37.1)	0 (0)	< 0.0001
		One (%)	14 (40)	17 (48.6)	19 (54.3)	13 (37.1)	
		Two (%)	3 (8.6)	8 (22.9)	3 (8.6)	13 (37.1)	
		≥ Two (%)	0 (0)	0 (0)	0 (0)	9 (25.7)	
	Fifth	No (%)	20 (57.1)	12 (34.3)	15 (42.9)	0 (0)	< 0.0001
		One (%)	13 (37.1)	17 (48.6)	19 (54.3)	9 (25.7)	
		Two (%)	2 (5.7)	6 (17.1)	1 (2.9)	16 (45.7)	
		≥ Two (%)	0 (0)	0 (0)	0 (0)	10 (28.6)	
Existing organ dysfunction	First, no (%)		30 (85.7)	29 (82.9)	31 (88.6)	30 (85.7)	0.926
	Second, no (%)		32 (91.4)	29 (82.9)	31 (88.6)	30 (85.7)	0.735
	Third, no (%)		31 (88.6)	29 (82.9)	31 (88.6)	30 (85.7)	0.880
	Fourth, no (%)		31 (88.6)	30 (85.7)	32 (91.4)	30 (85.7)	0.865
	Fifth, no (%)		33 (94.3)	32 (91.4)	34 (97.1)	30 (85.7)	0.327
Existing pulmonary dysfunction	First, yes (%)		19 (54.3)	23 (65.7)	24 (68.6)	19 (54.3)	0.477
	Second, yes (%)		13 (37.1)	20 (57.1)	22 (62.9)	19 (54.3)	0.160
	Third, yes (%)		10 (28.6)	18 (51.4)	20 (57.1)	19 (54.3)	0.066
	Fourth, yes (%)		7 (20)	16 (45.7)	19 (54.3)	19 (54.3)	0.010
	Fifth, yes (%)		3 (8.6)	13 (37.1)	17 (48.6)	19 (54.3)	< 0.0001
Smoking	First	No (%)	8 (22.9)	10 (28.6)	12 (34.3)	15 (42.9)	0.693
		< 1 P/M (%)	20 (57.1)	19 (54.3)	18 (51.4)	14 (40)	
		≥ 1 P/M (%)	7 (20)	6 (17.1)	5 (14.3)	6 (17.1)	
	Second	No (%)	18 (51.4)	11 (31.4)	14 (40)	15 (42.9)	0.423
		< 1 P/M (%)	17 (48.6)	22 (62.9)	20 (57.1)	17 (48.6)	
		≥ 1 P/M (%)	0 (0)	2 (5.7)	1 (2.9)	3 (8.6)	
	Third	No (%)	25 (71.4)	15 (42.9)	20 (57.1)	17 (48.6)	0.121
		< 1 P/M (%)	10 (28.6)	20 (57.1)	14 (40)	16 (45.7)	
		≥ 1 P/M (%)	0 (0)	0 (0)	1 (2.9)	2 (5.7)	
	Fourth	No (%)	30 (85.7)	21 (60)	26 (74.3)	17 (48.6)	0.019
		< 1 P/M (%)	5 (14.3)	14 (40)	8 (22.9)	16 (45.7)	
		≥ 1 P/M (%)	0 (0)	0 (0)	1 (2.9)	2 (5.7)	
	Fifth	No (%)	33 (94.3)	25 (71.4)	30 (85.7)	19 (54.3)	0.003
		< 1 P/M (%)	2 (5.7)	10 (28.6)	5 (14.3)	15 (42.9)	
		≥ 1 P/M (%)	0 (0)	0 (0)	0 (0)	1 (2.9)	

Group A = FCEM + CCM; Group B = FCEM; Group B = CCM; Group D = routine care.

*Chi-square test.

The odds ratio (OR) for GEE and Panel models indicated a decrease in coexisting illness for patients in intervention groups vs. controls, with group A < B < C < D, respectively. No significant association between existing organ dysfunction and intervention group was noted (*P* > 0.05), but the chance of existing pulmonary dysfunction was statistically lower for group A vs. controls (group D) based on GEE (OR: 0.38, *P* = 0.021) and Panel analysis (OR: 0.007, *P* = 0.027). No significant differences were noted for the groups B and C (P > 0.05). Although smoking was different in the fourth and fifth years of follow-up, only the GEE model indicated a significant difference between group A and controls (OR: 0.430, *P* = 0.04) (Supplementary file, Fig. 2).

### Health-related quality of life findings

Table 3 presented the results for HRQoL variable scores, including QoL, perceived stress, state anxiety, trait anxiety, Barthel index (BI), Kessler psychological distress scale (K10), six-minute walk test (6MWT), and a free walking index (WI) test. QoL improved via the study across all groups, with significantly greater improvements noted in the intervention groups (A, B, and C) compared to controls (61.3 ± 4.7 vs. 24 ± 6, *P* < 0.0001). The greatest benefit was observed in Group A that the mean score of QoL was significantly increased from 21 ± 2 to 86 ± 3 (*P* < 0.0001). GEE and Panel analysis models (Supplementary file, Table 3) indicated positive effects of the intervention on increasing BI via 5-year follow-up for intervention groups vs. control group (17 ± 1.8 vs. 10 ± 1.8, *P* < 0.0001). During the study, K10 scores decreased across all groups with significantly greater improvements noted in the intervention groups (A, B, and C) compared to controls (24 ± 6 vs. 33.5 ± 4, *P* < 0.0001), and the greatest benefit was observed in group A (23 ± 6 vs. 26 ± 5, *P* < 0.0001). However, state anxiety increased across intervention groups (A, B and C) as compared to group D, with the greatest increase belonging to group A (45 ± 7 vs. 80 ± 0.2, *P* < 0.0001). Trait anxiety remained stable throughout the study in groups B, C, and D (*P* > 0.05), while decreasing in group A (54.5 ± 4 vs. 54 ± 3, *P* = 0.042). 6MWT index increased for all groups during the 5-year follow-up. Significant improvement was observed for aggregate intervention groups (A, B, and C) compared with controls (518 ± 213 vs. 418 ± 260, *P* = 0.032); however, the greatest individual benefit was observed in group A (71 ± 5 vs. 533 ± 221, *P* < 0.0001). The WI also increased for all groups throughout follow-up. When comparing aggregate intervention groups (A, B and C) to controls, significant improvement was noted in the intervention cohort (*P* < 0.05). However, when individually comparing groups A, B, and C to group D, only group A was significantly better than controls (23,202 ± 8040 vs. 18,871 ± 8421, *P* = 0.025).

**Table 3:** Health quality of life variables over 5-year follow-up based on rehabilitation models in four groups of study.

Variables	Measurement	Group A	Group B	Group C	Group D	P-value*
BI	First (mean ± SD)	7.57 ± 0.85	7.75 ± 0.78	7.66 ± 0.87	7.91 ± 0.85	0.274
	Last (mean ± SD)	19.17 ± 0.92	15.89 ± 2.07	16.17 ± 2.47	9.49 ± 1.79	< 0.0001
K10	First (mean ± SD)	25.74 ± 5.44	26.74 ± 5.5	24.6 ± 4.21	26.37 ± 5.42	0.330
	Last (mean ± SD)	23.11 ± 6.14	25.86 ± 6.22	24.46 ± 4.87	33.51 ± 3.9	< 0.0001
Quality of life	First (mean ± SD)	21.4 ± 1.72	22.0 ± 1.83	22.14 ± 2.29	21.71 ± 2.22	0.433
	Last (mean ± SD)	85.74 ± 2.6	49.4 ± 5.95	48.6 ± 5.69	24.4 ± 5.84	< 0.0001
Anxiety state	First (mean ± SD)	45.37 ± 7.21	45.06 ± 6.23	44.37 ± 7.21	44.06 ± 6.23	0.838
	Last (mean ± SD)	79.97 ± 0.17	65.26 ± 8.07	62.31 ± 8.98	50.4 ± 7.22	< 0.0001
Anxiety trait	First (mean ± SD)	54.49 ± 4.36	53.43 ± 3.85	54.71 ± 4.17	53.54 ± 3.81	0.440
	Last (mean ± SD)	53.94 ± 3.55	54.97 ± 3.92	54.31 ± 4.78	56.03 ± 3.88	0.154
Stress	First (mean ± SD)	34.57 ± 3.83	33.49 ± 2.71	33.23 ± 1.97	33.91 ± 2.02	0.190
	Last (mean ± SD)	69.91 ± 0.51	61.43 ± 5.61	52.29 ± 5.26	46.26 ± 3.92	< 0.0001
6MWT	First (mean ± SD)	71.24 ± 5.31	67.12 ± 5.94	66.67 ± 6.25	73.8 ± 6.59	0.151
	Last (mean ± SD)	533.73 ± 221.76	509.86 ± 209.05	509.72 ± 210.88	417.74 ± 259.86	< 0.0001
WI	First (mean ± SD)	569.91 ± 42.47	536.97 ± 47.5	533.37 ± 49.97	590.4 ± 52.67	0.131
	Last (mean ± SD)	23,202.97 ± 8040.55	21,467.0 ± 7003.6	21,298.51 ± 6933.34	18,871.46 ± 8421.87	< 0.0001

Group 1 = FCEM + CCM; Group 2 = FCEM; Group 3 = CCM; Group 4 = Control.

BI: Barthel Index; K10: Kessler Psychological Distress Scale; 6MWT: six-minute walk test; WI: walking index.

*ANOVA.

**Figure 2:**
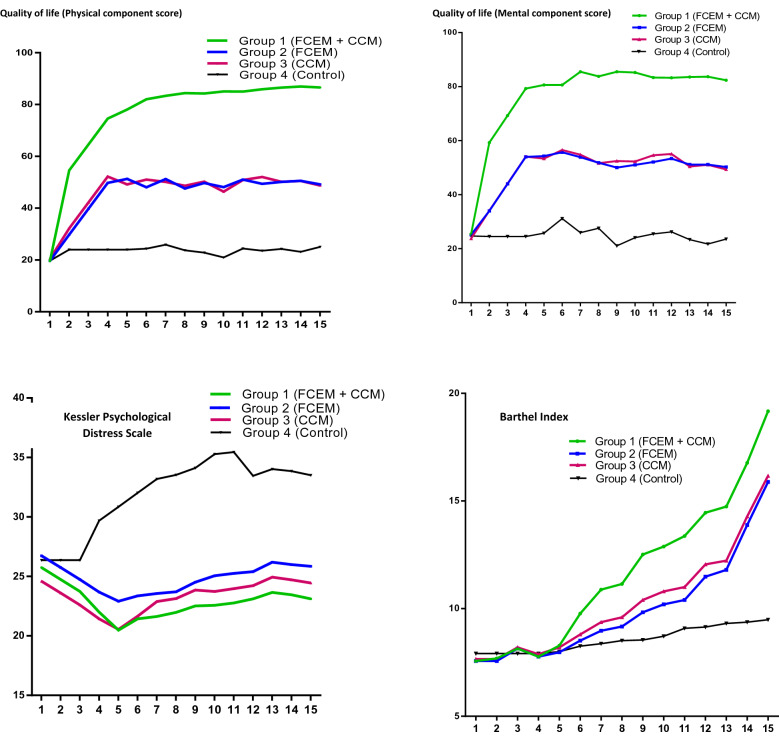
Repeated measurements of life satisfaction items during a 5-year follow-up. Horizontal axis (x-axis) represents a different time of measurement as follows; Number 1: pre-intervention, Number 2: 10-day post intervention, Number 3: 3-month post measurement 2, Numbers 4–9: following ups includes six 3-month periods (6, 9, 12, 15, 18, 21), Numbers 10–13: following ups includes four 3-month periods (27, 33, 39, 45), and Numbers 14–15: following ups includes two 12-month period (57, and 60) after intervention by deploying family centered empowerment model (FCEM) and continuous care model (CCM).

Pre- and post-intervention, as well as during a 5-year follow-up, repeated measurements of life satisfaction items were evaluated by soft outcome via two SF-36 questionnaire items (physical and mental components score) and two hard outcomes (K10) and (BI) in pre- and post-intervention and during the 5-year follow-up (Fig. 2).Besides, to determine the time of better rate of BI and K10 index over 5-year follow-up, we used a multiple Cox survival analysis (Supplementary file, Table 1). The results for BI indicated better scores for intervention groups, compared to controls as the reference group. In group A, the hazard ratio (HR) of the score of BI upper than 14 was (HR: 87.65, *P* < 0.0001) compared to controls (Supplementary file, Fig. 2a). Furthermore, the probability of a higher score of BI (> 14) increased for those who reported only one coexisting illness through the follow-up (HR: 2.045, *P* = 0.017). Besides, the HR of the lower K10 index was (HR: 2.179, *P* = 0.048), and (HR: 2.409, *P* = 0.009), in the first and second intervention groups, respectively. While it was not significant for third groups (HR: 0.615, *P* = 0.264), compared to controls (Supplementary file, Fig. 2b). Besides, BMI (*P* = 0.139) and Returned to work first year (*P* = 0.170) indicated no prognosis on KPDS.

### Pulmonary function findings

Table 4 shows the outcomes of pulmonary function testing on ARDS survivors during a 5-year period. Throughout the follow-up period, all groups' total lung capacity (TLC) rose. GEE and Panel analysis indicated that TLC improvement amongst combined intervention groups (A, B, and C) was significantly improved in comparison with controls (91 ± 7 vs. 83 ± 9, *P* < 0.0001). Individual improvements were noted for group A > B > C, respectively. The same improvement patterns were also observed for forced expiratory volume in 1 s (FEV_1_), forced vital capacity (FVC), and the diffusing capacity of lungs for carbon monoxide (DLCO). The ratio of FEV1 to FVC (FEV1/FVC), was another significant increased pulmonary function test which increased in all intervention groups, compared to controls (80 ± 10 vs. 73 ± 6, *P* < 0.0001), according to GEE and Panel analysis (Supplementary file, Table 4), but for total intervention groups, it was not significant (*P* = 0.109). Finally, RV test was decreased via the 5-year follow-up for intervention groups vs. controls and total intervention groups according to both models (*P* < 0.0001).

**Table 4:** Pulmonary function variables over 5-year follow-up based on rehabilitation models in four groups of study.

Variables	Measurement	Group 1	Group 2	Group 3	Group 4	P-value*
TLC	First (mean ± SD)	65.77 ± 1.06	65.31 ± 1.13	65.46 ± 1.17	65.57 ± 1.12	0.378
	Last (mean ± SD)	96.03 ± 6.12	94.37 ± 6.59	84.03 ± 8.29	82.6 ± 9.24	< 0.0001
DLCO	First (mean ± SD)	57.29 ± 1.27	57.11 ± 1.59	57.23 ± 1.06	57.14 ± 1.17	0.942
	Last (mean ± SD)	86.83 ± 12.36	84.8 ± 11.75	71.77 ± 6.69	68.94 ± 6.69	< 0.0001
FEV1	First (mean ± SD)	63.29 ± 1.15	63.57 ± 1.06	63.51 ± 0.78	63.26 ± 1.04	0.468
	Last (mean ± SD)	82.74 ± 10.18	80.46 ± 11.24	73.23 ± 6.38	70.86 ± 6.01	< 0.0001
FVC	First (mean ± SD)	64.54 ± 0.85	64.23 ± 1.16	64.03 ± 1.49	63.86 ± 1.35	0.119
	Last (mean ± SD)	92.00 ± 11.16	91.17 ± 11.41	79.6 ± 13.87	72.8 ± 8.85	< 0.0001
FEV1/FVC	First (mean ± SD)	67.69 ± 0.80	67.71 ± 0.83	67.63 ± 0.73	67.77 ± 0.60	0.879
	Last (mean ± SD)	85.31 ± 12.70	80.54 ± 12.48	73.71 ± 5.81	73.09 ± 5.18	< 0.0001
RV	First (mean ± SD)	127.86 ± 5.75	127.54 ± 5.56	128.8 ± 5.25	129.0 ± 5.64	0.635
	Last (mean ± SD)	79.57 ± 16.67	79.23 ± 14.36	97.37 ± 11.44	101.57 ± 13.18	< 0.0001

Group 1 = FCEM + CCM; Group 2 = FCEM; Group 3 = CCM; Group 4 = Control.

TLC: Total lung capacity; DLCO: Diffusing capacity of the lungs for carbon monoxide; FEV_1_: Forced expiratory volume in 1 s; FVC: Forced vital capacity; RV: Residual volume.

*ANOVA.

### SEM findings

SEM was performed to identify direct and indirect factors influencing outcomes in the patients experiencing. The normed χ^2^ was 1.57, indicating excellent fit. Moreover, the RMSEA = 0.064, IFI = 0.99, and CFI = 0.99 indicating that the model, including these factors performed better to describe the data. Therefore, the modified model fits the data (Supplementary file, Table 5).

Among many factors included in the SEM to evaluate the impact of clinical factors on ARDS patient outcomes, independent factors, including TLC, DLCO, FVC, FEV_1_, FEV_1_/FCV, WI, and (6MWT) were significantly increased by pooled intervention group (groups 1–3). While the effect of this variable decreased RV and K10.DLCO directly correlated with K10 (β = 0.208, *P* < 0.05), and K10 displayed a significant positive correlation with both WI and (6MWT). No other variables displayed significant direct effects. No variables displayed significant indirect effects amongst individual intervention groups. However, when pooling intervention groups (A, B, and C), DLCO was identified as a mediator through which WI and (6MWT) indirectly associated with increased K10 scores (Supplementary file, Fig. 3).

## Discussion

This study was conducted to determine the effect of FCEM and CCM rehabilitation techniques on pulmonary functions and HRQoL of ARDS survivors. According to the results, overall life satisfaction, including QoL, mental health (perceived stress and psychological distress), and physical health (physical state, activities of daily living, and exercise capacity), improved during the study in all groups, and significantly improvement was seen in the intervention groups compared to the control group, and the greatest benefit was observed in patients that used the mixture of FCEM and CCM rehabilitation techniques (group A). However, state anxiety increased among the intervention groups compared to the control group, and the highest increase was related to the first group that received FCEM and CCM programs, and trait anxiety was stable during the study in groups B and C as intervention groups and group D as the control group, while in the first group (group A) a decrease was observed. The stable situation of trait anxiety and the increasing rate of state anxiety can be a reflection of the patient's sense of empowerment in managing his or her own health. Besides, our interventions do not include forced treatment or interventions that can change the nature of patients' anxiety. Therefore, this level remained stable^14^. In terms of pulmonary function, measured factors, including TLC, FEV_1_, FVC, FEV_1_/FVC and DLCO increased and RV decreased in all groups throughout the 5-years follow-up. Analysis indicated that the pulmonary factors significantly improved among intervention groups compared to the controls.

A few studies reported the recovery of pulmonary functions, exercise capacity, and QoL in patients who survived ARDS20-22. A study by Hsieh et al.^11^, showed improvement in pulmonary function and exercise capacity in ARDS survivors of influenza A (H1N1) at 3 months after hospital discharge due to exercise, simple strength training, and respiratory exercise as SPR. Although, they reported the improvement of the QoL of these patients at 6 months after hospital discharge, even though there was no further improvement of their pulmonary functions and exercise capacity. Evidence suggests that lung volume shows a strong tendency to return to normal 3 to 6 months after the acute phase^20,21^. However, 6% to 43% of patients develop an obstructive pattern, and 15% to 58% develop a restrictive pattern in the first year of follow-up^22^. In addition, previous studies showed that 1 year after discharge, the 6-min walking distance has increased compared to the findings of immediate post-discharge period^9,23^.

To date, no studies are examining the effect of FCEM and CCM methods and comparing them to standard pulmonary rehabilitation on ARDS survivors. However, few studies were performed on the effects of these methods on the patients with other chronic illness^15–19^. The results of these studies about using an FCEM on caregivers and family members of patients with chronic illness, have been shown some improvement in the quality of life and mental health, which was consistent with our findings and reported the implementation of an FCEM intervention can reduce the burden of disease, and improve the mental and physical health of patients^24–26^. According to them, FCEM reduces the burden of care through its three axes: motivation, psychology (self-confidence, self-control, and self-efficacy), and problem-solving capacity. In terms of effect CCM, several studies showed that nurses could apply the CCM as an effective method to reduce risk factors and improve the lifestyle of patients with chronic disease30. However, a study conducted by Mohammadi et al.^27^, to determine the effect of using CCM at home on the QoL of patients with myocardial infarction (MI) showed that a rehabilitation program consisting of training sessions on MI and its complications, diet food, and medicine, risk factors, etc. at home did not have a significant effect on different dimensions of QoL of patients and there was no significant difference among the groups. These discrepancies could be related to sample size, patient follow-up period, and type of chronic disease.

In general, the present study results showed that in all groups, in the post-intervention phase, pulmonary function, exercise capacity (6MWD and WI), and HRQoL improved. These improvements were significantly higher in the intervention groups than in the control group. Among the intervention groups, the most improvement occurred in group “A” patients who used FCEM and CCM rehabilitation methods. The findings support the hypothesis that the synergistic effect of FCEM and CCM methods can significantly improve the patient's physical and mental function, reduce the burden of disease and improve the pulmonary function of ARDS survivors. CCM model's major purpose is to provide ongoing follow-up treatment in order to manage the illness and any potential consequences. Over the course of 12 weeks, four steps of identification, sensitization, control, and assessment are completed; the first two take 3 weeks while the other two take 9 weeks. The FCEM model's primary purpose, however, is to empower the family system and the patient as a whole in order to enhance their health. Perceived danger (severity and sensitivity), self-efficacy, self-esteem (confidence), and appraisal are the phases. As a result, the CCM model's basic and crucial concept of "follow-up and continuity of care" and the FCEM model's key concept of "the inseparable whole of the family and patient system" were merged; the synergistic effect of the two very important concepts mentioned had a dramatic effect on the first group's results when compared to the other two intervention groups (each model separately). Besides, encouraging the patient, understanding the patient more, reinforcing positive and feeling responsive via the simultaneous participants of family or friends (FCEM), and being in contact with a nurse (CCM), which creates a greater sense of support and satisfaction. This is reflected in the results of physical and mental health scores.

To our knowledge, this is the first research that evaluates and compares the synergistic effect of FCEM and CCM methods on pulmonary functions and HRQoL variables in ARDS survivors with standard pulmonary rehabilitation. Besides, this multi-center study was well-designed with a suitable sample size and 5-year follow-up. Nevertheless, this study had some limitations, which related to its nature. It was a randomized, controlled, and blinded prospective study, and 5 years of follow-up of patients was very difficult and in terms of the loss of follow-up has led to a decrease in sample size.

Finally, co-administration of FCEM and CCM may enhance pulmonary function as well as ARDS survivors' life satisfaction. As a consequence, ARDS survivors and their families are encouraged to participate in the empowerment paradigm, which should be carried out by nurses. Besides, further studies regarding the synergistic effect of FCEM and CCM models on the care performance of caregivers, as well as the pulmonary functions and QoL of ARDS survivors due to side effects, are suggested.

## Methods

### Trial design

This randomized controlled clinical trial was conducted to evaluate the effects of CCM or FCEM or both on the life satisfaction of ARDS survivors. From December 2009 to October June 2016, 140 ARDS survivors from mixed medical-surgical ICUs at four academic teaching hospitals in Tehran, Iran, were randomly assigned to one of three intervention groups (A, B, or C) or a control group (D). Pre- and post-interventions, pulmonary functions, and HRQoL status of patients in all groups were collected 48 times via clinical measurements and various questionnaires during 5 years of follow-up. All parts of the study were reviewed according to the Consolidated Standards of Reporting Trials (CONSORT) statement^28^. The protocol study was reviewed and approved by the Ethics Committees of Hamadan University of Medical Sciences (IR.UMSHA.REC.1400.604). On June 1, 2016, the experiment was registered with Clinicaltrials.gov (NCT02787720). On release from the ICU, the patient provided written permission. In circumstances where the patient lacked decision-making ability, surrogate consent from the patient's legal guardian or healthcare proxy was allowed. Furthermore, the research followed the guidelines of the Helsinki Declarations^29^.

### Eligibility criteria of participants

From December 2009 to June 2016, ICU patients with ARDS were screened for eligibility. The diagnosis of ARDS was based on the Berlin definition^1^, which was as follows; patients with a BMI < 40 suffering from ARDS with PaO_2_/FiO_2_ < 300 mmHg during mechanically ventilated (MV) with an expected duration of controlled MV of more than 24 h and ability to tolerate PEEP titration (up to 21 or 15 cmH_2_O). Besides, the patients were eligible to enroll in this study if they met the following criteria: (a) age ≥ 18 years, (b) able and willing to provide informed consent, (c) willingness of a family member or friend of patients to participate in the study, (d) has basic health literacy and can fill out questionnaire, and (e) full code status. Patients aged less than 18 years and more than 85 years, pregnant women, patients at end-stage medical condition, patients with a history of pulmonary rehabilitation, and patients with a history of neurological or psychiatric disorders were excluded from the study.

### Sample size, randomization and blinding

According to power estimates, each group would need 32 patient/family units to obtain a 95 percent confidence level and 90% power. Convenient sampling was used to enlist participants. In terms of sample loss, the sample size in each of the intervention and control groups was eventually determined to be 35. Eligible patients admitted to ICU were enrolled within 24 h and randomly assigned to one of three intervention groups (A, B, or C) or a control group (D). Group (A) who has received both FCEM and CCM programs via trainer. Group (B) received only FCEM via family member/friend as a trainer. Group (C) who received only CCM via researcher as trainer, and group (D) who used routine care as a control group. Randomization was achieved with a computer-generated random block design, which was drawn up by an expert statistician who had no clinical involvement in the trial before the beginning of study. Block randomization was accomplished using Random Allocation Software© (Informer Technologies, Inc., Madrid, Spain) by computer-generated random. Randomly allocated numbers were placed into sequential containers (i.e. ABCD, containers 1–4; BADC, containers 5–8, etc.) which were kept in a secure location until allocation consignment. The difference among groups was not disclosed, patients consented knowing that they were undergoing pulmonary rehabilitation (PR), but without knowing the details. The assignment was made through confidential communication between the patient's and a third party not involved in the recruitment process. Hence, patients and their family members, as well as data analyzers, were blinded to the assignment group and the differences among the groups.

### Intervention

The intervention package had three phases: pre-intervention, intervention, and post-intervention phases (Supplementary file, Fig. 1, and additional explanation about methods).

### Role of the designee and rehabilitation plan

Following informed permission, the selected family member or friend (hence referred to as designee) remained with the patient as a 'unit' throughout the trial. During stages 3 and 4, the designee attended the patient's educational sessions, with stage 2 being optional based on the designee's preferences. All patients had similar inpatient rehabilitation programs. For the patients in three intervention groups, outpatient rehab included daily exercise for 0–2 h/day. Exercise occurred between 8:00 and 10:00, and types included walking, jogging, bicycle, swimming, or other exercises according to patient preference or resource availability and confirmed by the multidisciplinary medical team.

### Data collection

Age, gender, body mass index (BMI), marital status, family number, household size, urban home location status, full- or part-time employment status, cause of ARDS, ICU, and hospital length of stay were collected as socio-demographic and clinical characteristics of patients. Initial illness severity was compared among groups using validated scales including Simplified Acute Physiology Score (SAPS)-III^30,31^, Sequential Organ Failure Assessment (SOFA)-II^32,33^, Acute Physiology and Chronic Health Evaluation (APACHE)-IV^34^, Lung Injury Score (LIS), and Multi Organ Dysfunction Score (MODS) at the first day of ICU admission^35^. Moreover, select treatment requirements included renal replacement therapy, paralytic treatment (> 1 bolus dose or continuous infusion), systemic stress-dose glucocorticoid treatment (e.g., Hydrocortisone 200 mg IVP × 1 then 100 mg IV three times daily for 5 days), tracheostomy placement, or use of high-frequency oxygenation technique including high-frequency oscillation ventilation (HFOV) and high-frequency percussive ventilation (HFPV). The high frequency techniques were specifically recorded as they are not part of routine ICU ventilator care, unlike Bi-level ventilation or airway pressure-release ventilation (APRV). Prone positioning is not routinely used in Iranian ICUs.

### Research instruments

Life satisfaction in all ARDS survivors was assessed via several questionnaires and tests in terms of physical and mental health as well as the quality of life index. Data collection tools consisted of hard outcomes and soft outcomes. Hard outcomes included the Barthel Index (BI) activities of daily living index, six-minute walk test (6MWT), free walking index (WI), and pulmonary function tests (PFT). Soft outcomes included the short-form health survey of quality of life (SF-36) questionnaire, the perceived stress (PSQ-14) questionnaire, state/trait anxiety, and the Kessler Psychological Distress Scale (K10). Both hard and soft outcomes, except the PFT, were evaluated 48 times over 5-year or 60 months as follows; in the pre-intervention as the baseline, monthly for 42 months continuously after the intervention, in the months 45, 48, 51, 54, 57, and 60 (explanation about each instrument are available in supplement file).

### Statistical analysis

The sample size in each group was calculated to be 32, with a confidence level of 95 percent and a test power of 80 percent, assuming that the measure of the impact of FCEM and CCM on parental burden of care (middle effect size) is at least d = 10. In terms of sample loss, the sample size in each of the intervention and control groups was eventually determined to be 35. In order to control dropouts in this trial, 140 participants were recruited. Statistical analysis was performed using IBM^®^ SPSS^®^ Statistics 21.0 (IBM Corp., Armonk, NY) and IBM^®^ SPSS^®^ AMOS™ 21.0 (IBM Corp., Armonk, NY). Discrete variables are expressed as counts and percentages. Power calculations determined that 32 patient/family units were needed in each group to achieve a 95% confidence level and a 90% power. Continuous variables are expressed as means and standard deviations. Analysis of variance (ANOVA) and Chi-Square analyses was used to compare numeric and discrete variables. Generalized Estimation Equations (GEE) and Panel Analysis were performed on longitudinal data, and the results were expressed as odds ratios (ORs). Multiple Cox proportional hazards regression was used to assess the effect of treatment groups on Barthel time and KPDS time. Structural Equation Modeling (SEM) (more information about SEM are available in Supplementary file) was performed to examine the effects of clinical factors on outcomes of acute respiratory distress syndrome, and the model was evaluated using the root mean square error of approximation (RMSEA), the normed fit index (NFI), and the goodness-of-fit index (GFI). Statistical significance was defined as p-value < 0.05.

### Ethical approval

The protocol study was reviewed and approved by the Ethics Committees of Hamadan University of Medical Sciences (IR.UMSHA.REC.1400.604). The trial was registered with Clinicaltrials.gov (NCT02787720) on 24/05/2016. In addition, the study was conducted according to the Helsinki Declarations guideline.

## Supplementary Information

Supplementary Information.

## Data Availability

All data collected and analyzed during the current study are available from the corresponding author on reasonable request.
