# Supplementary material for: Assessment of 5-year outcomes of life satisfaction in survivors after rehabilitation programs: a multicenter clinical trial
Source: Sci Rep. 2022 Jan 27;12:1497. doi: 10.1038/s41598-022-05355-z (PMC8795423; doi:10.1038/s41598-022-05355-z)
Supplement: Supplementary file 1 — Supplementary Information. [file 41598_2022_5355_MOESM1_ESM.docx]

Month 1 to month 42 (each month), and months 45, 48, 51, 54, 57, 60, 63, and 66)

Medical outcomes

In pre-intervention, measurement 2 (10-day post intervention), measurement 3 (three-month post measurement 2), and ten following ups includes six three-month periods (6, 9, 12, 15, 18, 21), four six-month periods (27, 33, 39, 45), and two twelve-month period (57, and 69) after intervention by deploying FCEM and CCM questionnaires.

**Instruments:** Quality-of-life (SF-36), Percieved stress (PSQ-14), State and Trait Anxiety Basthel Index (BI) Kessler Psychological Distress Scale (K10) Six-minute walk test (6MWT)

**Medical measures:** Physical examination, Pulmonary-function testing (PFT), Posterior-anterior and lateral (PA-Lat) chest radiography (CXR), Resting oximetry, a standardized 6MWT with continuous oximetry, free walking test as long as the stamina and capability of patient, Laboratory tests as indicated

3-5 group sessions

3-5 patients

3-5 hours

Stage 4: Evaluation

3-5 group sessions

3-5 patients

3-5 hours

Stage 3: Acceptance

3-5 group sessions

3-5 patients

3-5 hours

Stage 2: Expectations

3-5 group sessions

3-5 patients

45-50 min

Stage 1: Awareness and cognition

POST INTERVENTION

INTERVENTION

PRE INTERVENTION

Visit with phycologist, psychiatrist and neurologist

**4. Control(n=35)**

**3. Researcher at trainer with CCM (n=35)**

**2. Close friend as trainer with FCEM (n=35)**

**1. Family-centered empowerment model and Continuous care model (n=35)**

Random Allocation Software ©

(n=283)

Consulted with a nurse and specialists

(1) Age ≥18 years

(2) Informed consent

(3) Willingness of designated family member or friend to participate

(4) Has basic health literacy and can fill out questionnaire

(5) Admitted to the ICU

(6) Full code status (7) met ARDS criteria

Acute Respiratory Distress Symptom

Intensive Care Units (n=4)

2016

2009

Measures:

**Figure 1:** Three phases of study design

**Additional explanation about methods**

***Pre-intervention phase:*** Prior to ICU discharge in pre-intervention phase, life satisfaction in all ARDS survivors was assessed through several multiple questionnaires and tests in terms of physical and mental health as well as quality of life index. Short form health survey of quality of life (SF-36) questionnaire, perceived stress questionnaire (PSQ-14), state/trait anxiety questionnaire, and Kessler Psychological Distress Scale (K10) were used as soft outcomes. Hard outcomes includes Barthel Index (BI) activities of daily living, six-minute walk test (6MWT), and free walking index (WI) test.

***Intervention phase:*** In the intervention phase, group “A” as the first group of intervention groups received both FCEM and CCM programs with a focus on continuity and support from friends/family. The second group of intervention groups “group B” received only FCEM rehabilitation technique, with additional support from trainer. The third group of intervention groups “group C” received only CCM rehabilitation program, with additional support from researcher. While the control group “group D” received routine care without any external organization. The intervention had four stages; first stage was the awareness and cognition. The patient was evaluated for their insight into their perceived illness severity and perceived sensitivity, or the degree to which they felt threatened by their illness ^1^. This was performed by means of 3–5 group sessions in the pre-intervention phase. Group sessions included 3–5 patients and lasted for 45–60 min each. Session content included assessments of the participants’ psychological and physical conditions as well as their attitude towards the nature, definition, risk factors, symptoms, medical and nursing care, and complications resulting from ARDS. In a second stage, patients were assessed for their expectations over 3-5 one-hour sessions^1^. Groups of 3–4 patients shared and learned from each other under the moderation and guidance of the principal researcher. In a third stage, the degree of patient acceptance was assessed using an educational participation method in group discussion. Patients reached practical solutions through using the problem-solving findings of the previous stage. Fourth stage consisted of formative and summative evaluations^1^. All patients contact their nurse every 2 days to report problems or complications. A multidisciplinary medical team evaluated the patient as a group. Thus, patients were evaluated weekly for 30 days by a pulmonologist, intensivist, internist, psychologist, psychiatrist, physiotherapist and occupational therapist. At each visit, the patient was interviewed, underwent a physical examination, pulmonary-function tests (PFT), posterior-anterior and lateral chest radiography (following discharge), resting oximetry, a standardized 6MWT with continuous oximetry, a free walking index (WI), and laboratory tests. In addition, at any time, if experienced a problem or complication, patients can notify the intensivist, multidisciplinary medical team or their primary care provider. The aim of the formative evaluation was to encourage patients to internalize their locus of control by encouraging personal responsibility about issues of health and seeing his/her self-empowerment. Summative evaluations were performed to evaluate the influence of the intervention on the study's medical outcomes.

***Post-intervention phase:*** All patients were followed for 5-year and their outcomes were assessed 48 times during 5-year or 60 months as follows; in the pre-intervention as baseline, monthly for 42 months continuously post-intervention, in months 45, 48, 51, 54, 57, and 60. During the 60-month follow-up period, patients attended a total of 56 support group webinars addressing topics including returning to work, intimate relationships, nutrition, sleep hygiene, tobacco use, exercise, and leisure activities as well as testing and laboratory issues. Follow-up interviews were conducted during home visits, when available, or with the assistance of telephone, Skype©, Viber©, WhatsApp© and social media options (e.g. Facebook©) according to patient preference.

***Role of the Designee:*** Recall that stage 2 deals with patient expectations, stage 3 with patient acceptance and problem-solving, and stage 4 with evaluations and internalizing his/her locus of control. The designee and the patient attended the same sessions, and studied the same learning materials. Up to eight family members were allowed to join in the educational sessions if requested. In stage 3, the designee was charged with learning and reinforcing educational material with the patient. In stage 4, when instructed by study investigators, the designee would administer the KAP assessments to the patient. In addition to scores, the designee would provide additional information on the patient’s home situation and current condition. Patients were assigned a code, and de-identified data were transmitted from the designee to investigators either by encrypted email, telephone, mail or in person.

***Rehabilitation plan:*** Daily exercise was supervised by designated family members. Investigators randomly attended sessions in an unannounced fashion. Although not routinely involved, multidisciplinary medical team consultations were available on investigator request. Exercise data were independently collected from the patient and their designated family member on a weekly basis (κ statistic=0.94). To measure the patients’ walking distance, investigators provided them with a Fitbit® (Fitbit, San Francisco, USA). Routine care included education on smoking cessation and education on food selection. Patients were provided printed materials, with dietician assessments available on request. Patients in the control group received the same education and printed materials during their inpatient course. Patients exercised daily, at any time, for ≤2 h according to patient tolerance. Sessions were supervised by family members. Investigators did not attend sessions. Exercise data were independently collected from the patient and their designated family member on a weekly basis (κ statistic=0.22). Walking distance was measured using the Fitbit. Routine care included education on smoking cessation and education on food selection. Patients were provided printed materials, with dietician assessments available on request.

**Research Instruments**

***SF-36 questionnaire:*** The questionnaire consists of eight domains including physical functioning, physical role limitation, social functioning, bodily pain, mental health, mental role limitation, vitality and general health. Scoring of each domain was calculated independently and scores ranged from 0 (the worst) to 100 (the best)^2, 3^. The SF-36 questionnaire is a validated tool, whose reliability in this study was assessed by test–retest and Cronbach’s alpha, 0.93 and 0.94, respectively ^4, 5^.

***The 14-item Perceived Stress Questionnaire (PSQ-14):*** Overall stress was measured using the validated 14-item perceived stress questionnaire (PSQ-14)^6^.PSQ-14 scores are obtained by reversing the scores on the seven positive items, including 0=4, 1=3, 2=2, 3=1 and 4=0, then, summing across all 14 items. Scores range from 14 to 70 ^7^.The reliability of thePSQ-14 was assessed in this study by test-retest and Cronbach’s alpha, 0.95 and 0.96, respectively ^8^.

The State/trait Anxiety questionnaire: Patient anxiety was assessed through the State-Trait Anxiety questionnaire^9^. This validated tool has 20 items for assessing trait anxiety and state anxiety, respectively. All items are rated on a four-point scale ranging from very low (1 point) to very high (4 points). Higher scores indicate greater anxiety ^1^. The reliability of the State-Trait Anxiety questionnaire was assessed in this study by test-retest and Cronbach’s alpha, 0.92 and 0.94, respectively ^10^.

***Barthel Index (BI) of Activities of Daily Living:*** The BI is an ordinal scale used to measure ADL’s. It uses 10 variables describing ADL and mobility ^11^. Scores range from 0 to 2 or 3 for each item. Total possible scores range from 0–20, with lower scores indicating increased disability. If used to measure improvement after rehabilitation, changes of more than two points in the total score reflect a probable genuine change, and change on one item from fully dependent to independent is also likely to be reliable. The reliability of the BI questionnaire was assessed in this study by test-retest and Cronbach’s alpha, 0.91 and 0.92 respectively ^12^.

***Kessler Psychological Distress Scale (K10):*** The questionnaire is a 10-item assessment intended to yield a global measure of distress based on questions about anxiety and depressive symptoms that a person has experienced in the most recent four-week period ^13^. All items are rated on a 5-point scale ranging from “none” (1 point) to “all” of the time (5 points). The maximum score is 50 (severe distress) and the minimum is 10 (no distress) ^14^. The reliability of the K10 questionnaire was assessed in this study by test–retest and Cronbach’s alpha, 0.93 and 0.92 respectively ^15^.

***Pulmonary function tests (PFT):*** PFT was conducted twice for all patients, once before the intervention and once after the intervention. PFT was performed by spirometry (which assesses static and dynamic pulmonary volumes) and diffusion capacity (which assesses the capacity for gas exchange through the alveolar barrier) techniques to assess pulmonary function in this study. All tests were proceeded according to American Thoracic Society guidelines, standardization of Spirometry 2019 update ^16^. The expiratory volume in the first second (FEV_1_) and vital capacity (FVC) during forced expiration were recorded. Total lung capacity (TLC) and residual volume (RV) were measured using the plethysmography method, and diffusing capacity of the lung for carbon monoxide (DLCO) was measured using the single breath- hold method ^17^.

***Structural equation modeling (SEM)***: It was used to assess for direct and indirect inter-variable associations (walking index that was checked every month) through mediators. Pulmonary factors including total lung capacity (TLC), diffusing capacity of the lungs for carbon monoxide (DLCO), residual volume (RV), forced vital capacity (FVC), forced expiratory volume in 1 second (FEV1), Kessler psychological distress scale (K10), and Barthel Index (BI) were added in the independent variable list. Descriptive statistics were used to summarize the data. Pearson correlation was employed to investigate the association between independent variables and other outcomes. SEM was used to find direct and indirect associations between independent variables and other outcomes. Since outcomes were not normally distributed (Kolmogorov-Smirnov *P*<0.05), the SEM model was estimated using Asymptotically Distribution-Free estimates (ADF). To assess model fit, indices including (normed fit index (NFI), incremental fit index (IFI), Tucker-Lewis index (TLI), and comparative fit index (CFI), root mean square error of approximation (RMSEA), and chi T square/DF ratio (χ^2^/DF) were calculated as goodness-of-fit index(GFI). Direct association was reported in standardized parameters, whereas indirect association was reported through the mediator’s effects. If both direct and indirect effects were significant for a proposed covariate, the total association was estimated by sum-of-two effects. Modeling and statistics were performed using IBM® SPSS® AMOS™ 21 (IBM Corp., Armonk, NY) ^18^.

**Table1:** Multiple Cox survival analysis of better scores of Barthel index (BI) and Kessler psychological distress scale (KPDS) over five years

|  | **Measurement** | **HR** | **P value** | **Lower** | **Upper** |
| --- | --- | --- | --- | --- | --- |
| **Barthe Index** | **Group 4** | **Reference** | | | |
|  | **Group 1** | 87.652 | **<0.0001** | 20.575 | 373.406 |
|  | **Group 2** | 28.462 | **<0.0001** | 6.770 | 119.654 |
|  | **Group 3** | 29.226 | **<0.0001** | 6.983 | 122.317 |
|  | **Coexisting Illness First year, (No)** | **Reference** | | | |
|  | **Coexisting Illness First year, (One)** | 2.045 | **0.017** | 1.139 | 3.672 |
|  | **Coexisting Illness First year, (Two)** | 1.062 | **0.856** | 0.555 | 2.030 |
|  | **Coexisting Illness First year, (>Two)** | 1.070 | **0.803** | 0.628 | 1.825 |
| **KPDS Index** | **Group 4** | **Reference** | | | |
|  | **Group 1** | 2.179 | **0.048** | 1.103 | 4.454 |
|  | **Group 2** | 2.409 | **0.009** | 1.196 | 4.364 |
|  | **Group 3** | 0.615 | **0.264** | 0.258 | 1.416 |
|  | **Gender** | 1.322 | **0.233** | 0.824 | 2.061 |
|  | **BMI** | 0.845 | **0.139** | 0.896 | 1.071 |
|  | **Returned to work first year** | 0.684 | **0.170** | 0.418 | 1.232 |

**Table 2:** Changes in demographic and clinical characteristics over five-year follow-up based on rehabilitation models in four groups of study

| **P-Value***** | **Group D** | **Group C** | **Group B** | **Group A** | **Years** | | **Variables** |
| --- | --- | --- | --- | --- | --- | --- | --- |
| **<0.0001** | 0 (0) | 18 (51.4) | 14 (40) | 27 (77.1) | **First,** Yes (%) | | **Returned to work** |
| **<0.0001** | 3 (8.6) | 24 (68.6) | 23 (65.7) | 30 (85.7) | **Second,** Yes (%) | |  |
| **<0.0001** | 5 (14.3) | 28 (80) | 26 (74.3) | 32 (91.4) | **Third,** Yes (%) | |  |
| **<0.0001** | 7 (20) | 33 (94.3) | 30 (85.7) | 33 (94.3) | **Fourth,** Yes (%) | |  |
| **<0.0001** | 11 (31.4) | 34 (97.1) | 32 (91.4) | 33 (94.3) | **Fifth,** Yes (%) | |  |
|  | **Reference** | 18.18 **(<0.0001)** | 12.20 **(<0.0001)** | 38.46 **(<0.0001)** | **OR (P - Value) *** | |  |
|  | **Reference** | 240.78 **(<0.0001)** | 124.68 **(<0.0001)** | 806.46 **(<0.0001)** | **OR (P - Value) **** | |  |
|  | **Reference** | 19.23 **(<0.0001) &**319.34 **(<0.0001)** | | | **OR (P - Value) * & **** | |  |
| **0.006** | 0 (0) | 11 (31.4) | 7 (20) | 8 (22.9) | **First,** Yes (%) | | **Returned to Original work** |
| **<0.0001** | 0 (0) | 16 (45.7) | 13 (37.1) | 19 (54.3) | **Second,** Yes (%) | |  |
| **<0.0001** | 0 (0) | 19 (54.3) | 18 (51.4) | 22 (62.9) | **Third,** Yes (%) | |  |
| **<0.0001** | 1 (2.9) | 26 (74.3) | 23 (65.7) | 27 (77.1) | **Fourth,** Yes (%) | |  |
| **<0.0001** | 5 (14.3) | 29 (82.9) | 25 (71.4) | 31 (88.6) | **Fifth,** Yes (%) | |  |
|  | **Reference** | 38.44 **(<0.0001)** | 27.22 **(<0.0001)** | 44.32 **(<0.0001)** | **OR (P - Value) *** | |  |
|  | **Reference** | 56.53 **(<0.0001)** | 37.76 **(<0.0001)** | 69.06 **(<0.0001)** | **OR (P - Value) **** | |  |
|  | **Reference** | 30.69 **(<0.0001) &**51.24 **(<0.0001)** | | | **OR (P - Value) * & **** | |  |
| **0.963** | 10 (28.6) | 9 (25.7) | 6 (17.1) | 9 (25.7) | No (%) | **First** | **Coexisting Illness** |
|  | 6 (17.1) | 7 (20) | 7 (20) | 6 (17.1) | One (%) |  |  |
|  | 12 (34.3) | 12 (34.3) | 16 (45.7) | 11 (31.4) | Two (%) |  |  |
|  | 7 (20) | 7 (20) | 6 (17.1) | 9 (25.7) | ≥Two (%) |  |  |
| **0.001** | 4 (11.4) | 9 (25.7) | 7 (20) | 12 (34.3) | No (%) | **Second** |  |
|  | 13 (37.1) | 17 (48.6) | 14 (40) | 13 (37.1) | One (%) |  |  |
|  | 11 (31.4) | 9 (25.7) | 14 (40) | 10 (28.6) | Two (%) |  |  |
|  | 7 (20) | 0 (0) | 0 (0) | 0 (0) | ≥Two (%) |  |  |
| **<0.0001** | 1 (2.9) | 12 (34.3) | 9 (25.7) | 14 (40) | No (%) | **Third** |  |
|  | 13 (37.1) | 17 (48.6) | 15 (42.9) | 15 (42.9) | One (%) |  |  |
|  | 15 (42.9) | 6 (17.1) | 11 (31.4) | 6 (17.1) | Two (%) |  |  |
|  | 6 (17.1) | 0 (0) | 0 (0) | 0 (0) | ≥Two (%) |  |  |
| **<0.0001** | 0 (0) | 13 (37.1) | 10 (28.6) | 18 (51.4) | No (%) | **Fourth** |  |
|  | 13 (37.1) | 19 (54.3) | 17 (48.6) | 14 (40) | One (%) |  |  |
|  | 13 (37.1) | 3 (8.6) | 8 (22.9) | 3 (8.6) | Two (%) |  |  |
|  | 9 (25.7) | 0 (0) | 0 (0) | 0 (0) | ≥Two (%) |  |  |
| **<0.0001** | 0 (0) | 15 (42.9) | 12 (34.3) | 20 (57.1) | No (%) | **Fifth** |  |
|  | 9 (25.7) | 19 (54.3) | 17 (48.6) | 13 (37.1) | One (%) |  |  |
|  | 16 (45.7) | 1 (2.9) | 6 (17.1) | 2 (5.7) | Two (%) |  |  |
|  | 10 (28.6) | 0 (0) | 0 (0) | 0 (0) | ≥Two (%) |  |  |
|  | **Reference** | 0.22 **(<0.0001)** | 0.32 **(0.002)** | 0.19 **(<0.0001)** | **OR (P - Value) *** | |  |
|  | **Reference** | 0.034 **(0.005)** | 0.13 **(0.084)** | 0.008 **(<0.0001)** | **OR (P - Value) **** | |  |
|  | **Reference** | 0.23 **(<0.0001) &**0.033 **(<0.0001)** | | | **OR (P - Value) * & **** | |  |
| **0.926** | 30 (85.7) | 31 (88.6) | 29 (82.9) | 30 (85.7) | **First,** No (%) | | **Existing Organ dysfunction** |
| **0.735** | 30 (85.7) | 31 (88.6) | 29 (82.9) | 32 (91.4) | **Second,** No (%) | |  |
| **0.880** | 30 (85.7) | 31 (88.6) | 29 (82.9) | 31 (88.6) | **Third,** No (%) | |  |
| **0.865** | 30 (85.7) | 32 (91.4) | 30 (85.7) | 31 (88.6) | **Fourth,** No (%) | |  |
| **0.327** | 30 (85.7) | 34 (97.1) | 32 (91.4) | 33 (94.3) | **Fifth,** No (%) | |  |
|  | **Reference** | 0.604 **(0.480)** | 1.05 **(0.94)** | 0.69 **(0.58)** | **OR (P - Value) *** | |  |
|  | **Reference** | 0.25 **(0.580)** | 0.80 **(0.872)** | 0.51 **(0.676)** | **OR (P - Value) **** | |  |
|  | **Reference** | 1.452 **(0.504) &**0.27 **(0.374)** | | | **OR (P - Value) * & **** | |  |
| **0.477** | 19 (54.3) | 24 (68.6) | 23 (65.7) | 19 (54.3) | **First,** Yes (%) | | **Existing pulmonary dysfunction** |
| **0.160** | 19 (54.3) | 22 (62.9) | 20 (57.1) | 13 (37.1) | **Second,** Yes (%) | |  |
| **0.066** | 19 (54.3) | 20 (57.1) | 18 (51.4) | 10 (28.6) | **Third,** Yes (%) | |  |
| **0.010** | 19 (54.3) | 19 (54.3) | 16 (45.7) | 7 (20) | **Fourth,** Yes (%) | |  |
| **<0.0001** | 19 (54.3) | 17 (48.6) | 13 (37.1) | 3 (8.6) | **Fifth,** Yes (%) | |  |
|  | **Reference** | 1.19 (**0.708)** | 0.89 **(0.796)** | 0.38 **(0.021)** | **OR (P - Value) *** | |  |
|  | **Reference** | 2.78 **(0.689)** | 0.44 **(0.763)** | 0.007 **(0.027)** | **OR (P - Value) **** | |  |
|  | **Reference** | 0.75 **(0.436) &**0.12 **(0.475)** | | | **OR (P - Value) * & **** | |  |
| **0.693** | 15 (42.9) | 12 (34.3) | 10 (28.6) | 8 (22.9) | No (%) | **First** | **Smoking** |
|  | 14 (40) | 18 (51.4) | 19 (54.3) | 20 (57.1) | < 1 P/M (%) |  |  |
|  | 6 (17.1) | 5 (14.3) | 6 (17.1) | 7 (20) | ≥ 1 P/M (%) |  |  |
| **0.423** | 15 (42.9) | 14 (40) | 11 (31.4) | 18 (51.4) | No (%) | **Second** |  |
|  | 17 (48.6) | 20 (57.1) | 22 (62.9) | 17 (48.6) | < 1 P/M (%) |  |  |
|  | 3 (8.6) | 1 (2.9) | 2 (5.7) | 0 (0) | ≥ 1 P/M (%) |  |  |
| **0.121** | 17 (48.6) | 20 (57.1) | 15 (42.9) | 25 (71.4) | No (%) | **Third** |  |
|  | 16 (45.7) | 14 (40) | 20 (57.1) | 10 (28.6) | < 1 P/M (%) |  |  |
|  | 2 (5.7) | 1 (2.9) | 0 (0) | 0 (0) | ≥ 1 P/M (%) |  |  |
| **0.019** | 17 (48.6) | 26 (74.3) | 21 (60) | 30 (85.7) | No (%) | **Fourth** |  |
|  | 16 (45.7) | 8 (22.9) | 14 (40) | 5 (14.3) | < 1 P/M (%) |  |  |
|  | 2 (5.7) | 1 (2.9) | 0 (0) | 0 (0) | ≥ 1 P/M (%) |  |  |
| **0.003** | 19 (54.3) | 30 (85.7) | 25 (71.4) | 33 (94.3) | No (%) | **Fifth** |  |
|  | 15 (42.9) | 5 (14.3) | 10 (28.6) | 2 (5.7) | < 1 P/M (%) |  |  |
|  | 1 (2.9) | 0 (0) | 0 (0) | 0 (0) | ≥ 1 P/M (%) |  |  |
|  | **Reference** | 0.647 **(0.300)** | 1.036 **(0.933)** | 0.430 **(0.04)** | **OR (P - Value) *** | |  |
|  | **Reference** | 0.36 **(0.219)** | 0.97 **(0.969)** | 0.25 **(0.09)** | **OR (P - Value) **** | |  |
|  | **Reference** | 0.676 **(0.27) &**0.44 **(0.234)** | | | **OR (P - Value) * & **** | |  |

Group A = FCEM + CCM; Group B = FCEM; Group B = CCM; Group D = routine care; *Generalized Estimation Equations (GEE); **Panel (Adjusted); *** Chi-square

**Table 3:** Health quality of life variables over five-year follow-up based on rehabilitation models in four groups of study

|  | **Measurement** | **Group A** | **Group B** | **Group C** | **Group D** | **P-Value ***** |
| --- | --- | --- | --- | --- | --- | --- |
| **BI** | **First** (Mean ±SD) | 7.57±0.85 | 7.75±0.78 | 7.66±0.87 | 7.91±0.85 | **0.274** |
|  | **Last** (Mean ±SD) | 19.17±0.92 | 15.89±2.07 | 16.17±2.47 | 9.49±1.79 | **<0.0001** |
|  | **B (P - Value) *** | 3.806**(<0.0001)** | 2.113 **(<0.0001)** | 2.386 **(<0.0001)** | **Reference** |  |
|  | **B (P - Value) **** | 3.11 **(<0.0001)** | 1.38 **(<0.0001)** | 1.72 **(<0.0001)** | **Reference** |  |
|  | **B (P - Value) * & **** | 2.792 **(<0.0001) &** 2.07 **(<0.0001)** | | | **Reference** |  |
| **K10** | **First** (Mean ±SD) | 25.74±5.44 | 26.74±5.5 | 24.6±4.21 | 26.37±5.42 | **0.330** |
|  | **Last** (Mean ±SD) | 23.11±6.14 | 25.86±6.22 | 24.46±4.87 | 33.51±3.9 | **<0.0001** |
|  | **B (P - Value) *** | -6.076 **(<0.0001)** | -4.186 **(<0.0001)** | -5.913 **(<0.0001)** | **Reference** |  |
|  | **B (P - Value) **** | -9.01 **(<0.0001)** | -7.03 **(<0.0001)** | -8.52 **(<0.0001)** | **Reference** |  |
|  | **B (P - Value) * & **** | -5.386 **(<0.0001) &** -8.18 **(<0.0001)** | | | **Reference** |  |
| **Quality of life** | **First** (Mean ±SD) | 21.4±1.72 | 22.0±1.83 | 22.14±2.29 | 21.71±2.22 | **0.433** |
|  | **Last** (Mean ±SD) | 85.74±2.6 | 49.4±5.95 | 48.6±5.69 | 24.4±5.84 | **<0.0001** |
|  | **B (P - Value) *** | 53.108 **(<0.0001)** | 23.008 **(<0.0001)** | 23.594 **(<0.0001)** | **Reference** |  |
|  | **B (P - Value) **** | 57.36 **(<0.0001)** | 24.15 **(<0.0001)** | 24.95 **(<0.0001)** | **Reference** |  |
|  | **B (P - Value) * & **** | 32.294 **(<0.0001) &**35.49 **(<0.0001)** | | | **Reference** |  |
| **Anxiety-State** | **First** (Mean ±SD) | 45.37±7.21 | 45.06±6.23 | 44.37±7.21 | 44.06±6.23 | **0.838** |
|  | **Last** (Mean ±SD) | 79.97±0.17 | 65.26±8.07 | 62.31±8.98 | 50.4±7.22 | **<0.0001** |
|  | **B (P - Value) *** | 16.957 **(<0.0001)** | 8.593 **(<0.0001)** | 6.785 **(<0.0001)** | **Reference** |  |
|  | **B (P - Value) **** | 20.39 **(<0.0001)** | 10.1 **(<0.0001)** | 8.31 **(<0.0001)** | **Reference** |  |
|  | **B (P - Value) * & **** | 10.607 **(<0.0001) &** 12.93 **(<0.0001)** | | | **Reference** |  |
| **Anxiety-Trait** | **First** (Mean ±SD) | 54.49±4.36 | 53.43±3.85 | 54.71±4.17 | 53.54±3.81 | **0.440** |
|  | **Last** (Mean ±SD) | 53.94±3.55 | 54.97±3.92 | 54.31±4.78 | 56.03±3.88 | **0.154** |
|  | **B (P - Value) *** | -1.199 (0.054) | -.803 (0.217) | -.367 (.574) | **Reference** |  |
|  | **B (P - Value) **** | -1.32 (0.042) | -0.84 (0.193) | -.39 (0.553) | **Reference** |  |
|  | **B (P - Value) * & **** | -.789 **(0.135) &**-.85 **(0.109)** | | | **Reference** |  |
| **Stress** | **First** (Mean ±SD) | 34.57±3.83 | 33.49±2.71 | 33.23±1.97 | 33.91±2.02 | **0.190** |
|  | **Last** (Mean ±SD) | 69.91±0.51 | 61.43±5.61 | 52.29±5.26 | 46.26±3.92 | **<0.0001** |
|  | **B (P - Value) *** | 13.914 **(<0.0001)** | 8.230 **(<0.0001)** | 3.294 **(<0.0001)** | **Reference** |  |
|  | **B (P - Value) **** | 16.74 **(<0.0001)** | 9.61 **(<0.0001)** | 4.29 **(<0.0001)** | **Reference** |  |
|  | **B (P - Value) * & **** | 8.317 **(<0.0001) &**10.22 **(<0.0001)** | | | **Reference** |  |
| **6MWT** | **First** (Mean ±SD) | 71.24±5.31 | 67.12±5.94 | 66.67±6.25 | 73.8±6.59 | **0.151** |
|  | **Last** (Mean ±SD) | 533.73±221.76 | 509.86±209.05 | 509.72±210.88 | 417.74±259.86 | **<0.0001** |
|  | **B (P - Value) *** | 306.876**(<0.0001)** | 250.934 **(<0.0001)** | 263.644 **(<0.0001)** | **Reference** |  |
|  | **B (P - Value) **** | 2365.4 (0.021) | 1471.73(0.151) | 1519.47(0.139) | **Reference** |  |
|  | **B (P - Value) * & **** | 273.562 **(<0.0001) &**1785.533 **(0.032)** | | | **Reference** |  |
| **WI** | **First** (Mean ±SD) | 569.91±42.47 | 536.97±47.5 | 533.37±49.97 | 590.4±52.67 | **0.131** |
|  | **Last** (Mean ±SD) | 23202.97±8040.55 | 21467.0±7003.6 | 21298.51±6933.34 | 18871.46±8421.87 | **<0.0001** |
|  | **B (P - Value) *** | 11177.608 **(<0.0001)** | 7017.395 **(<0.0001)** | 7495.505 **(<0.0001)** | **Reference** |  |
|  | **B (P - Value) **** | 65.46 (0.030) | 48.46 (0.108) | 51.10 (0.091) | **Reference** |  |
|  | **B (P - Value) * & **** | 8544.441 **(<0.0001) &**55.004 **(0.025)** | | | **Reference** |  |

Group 1 = FCEM + CCM; Group 2 = FCEM; Group 3 = CCM; Group 4 = Control, Abbreviations; BI: Barthel Index; K10: Kessler Psychological Distress Scale; 6MWT: six-minute walk test; WI: walking index; * Generalized Estimation Equations (GEE); **Panel (Adjusted); *** ANOVA

**Table 4:** Pulmonary function variables over five-year follow-up based on rehabilitation models in four groups of study

|  | **Measurement** | **Group 1** | **Group 2** | **Group 3** | **Group 4** | **P-Value ***** |
| --- | --- | --- | --- | --- | --- | --- |
| **TLC** | **First** (Mean ±SD) | 65.77±1.06 | 65.31±1.13 | 65.46±1.17 | 65.57±1.12 | **0.378** |
|  | **Last** (Mean ±SD) | 96.03±6.12 | 94.37±6.59 | 84.03±8.29 | 82.6±9.24 | **<0.0001** |
|  | **B (P - Value) *** | 9.528 **(<0.0001)** | 9.020 **(<0.0001)** | 8.388 **(<0.0001)** | **Reference** |  |
|  | **B (P - Value) **** | 5.97 **(<0.0001)** | 4.76 **(<0.0001)** | 1.74 **(<0.0001)** | **Reference** |  |
|  | **B (P - Value) * & **** | 8.979 **(<0.0001) &** 3.30 **(<0.0001)** | | | **Reference** |  |
| **DLCO** | **First** (Mean ±SD) | 57.29±1.27 | 57.11±1.59 | 57.23±1.06 | 57.14±1.17 | **0.942** |
|  | **Last** (Mean ±SD) | 86.83±12.36 | 84.8±11.75 | 71.77±6.69 | 68.94±6.69 | **<0.0001** |
|  | **B (P - Value) *** | 16.934 **(<0.0001)** | 4.230 **(<0.0001)** | 5.115 **(<0.0001)** | **Reference** |  |
|  | **B (P - Value) **** | 10.96 **(<0.0001)** | 9.88 **(<0.0001)** | 1.42 **(<0.0001)** | **Reference** |  |
|  | **B (P - Value) * & **** | 8.450 **(<0.0001) &** 7.42 **(<0.0001)** | | | **Reference** |  |
| **FEV_1_** | **First** (Mean ±SD) | 63.29±1.15 | 63.57±1.06 | 63.51±0.78 | 63.26±1.04 | **0.468** |
|  | **Last** (Mean ±SD) | 82.74±10.18 | 80.46±11.24 | 73.23±6.38 | 70.86±6.01 | **<0.0001** |
|  | **B (P - Value) *** | 9.266 **(<0.0001)** | 4.274 **(<0.0001)** | 2.681 **(<0.0001)** | **Reference** |  |
|  | **B (P - Value) **** | 7.03 **(<0.0001)** | 5.86 **(<0.0001)** | 1.38 **(<0.0001)** | **Reference** |  |
|  | **B (P - Value) * & **** | 5.350 **(<0.0001) &** 4.75 **(<0.0001)** | | | **Reference** |  |
| **FVC** | **First** (Mean ±SD) | 64.54±0.85 | 64.23±1.16 | 64.03±1.49 | 63.86±1.35 | **0.119** |
|  | **Last** (Mean ±SD) | 92.00±11.16 | 91.17±11.41 | 79.6±13.87 | 72.8±8.85 | **<0.0001** |
|  | **B (P - Value) *** | 16.533 **(<0.0001)** | 9.931 **(<0.0001)** | 8.262 **(<0.0001)** | **Reference** |  |
|  | **B (P - Value) **** | 11.01 **(<0.0001)** | 10.11 **(<0.0001)** | 3.2 **(<0.0001)** | **Reference** |  |
|  | **B (P - Value) * & **** | 11.579 **(<0.0001) &** 8.11 **(<0.0001)** | | | **Reference** |  |
| **FEV1FVC** | **First** (Mean ±SD) | 67.69±0.80 | 67.71±0.83 | 67.63±0.73 | 67.77±0.60 | **0.879** |
|  | **Last** (Mean ±SD) | 85.31±12.70 | 80.54±12.48 | 73.71±5.81 | 73.09±5.18 | **<0.0001** |
|  | **B (P - Value) *** | 10.314 **(<0.0001)** | 1.222 **(<0.0001)** | 1.889 **(<0.0001)** | **Reference** |  |
|  | **B (P - Value) **** | 6.37 **(<0.0001)** | 3.94 **(<0.0001)** | 0.26 **(<0.0001)** | **Reference** |  |
|  | **B (P - Value) * & **** | 4.490 **(0.135) &** 3.52 **(0.109)** | | | **Reference** |  |
| **RV** | **First** (Mean ±SD) | 127.86±5.75 | 127.54±5.56 | 128.8±5.25 | 129.0±5.64 | **0.635** |
|  | **Last** (Mean ±SD) | 79.57±16.67 | 79.23±14.36 | 97.37±11.44 | 101.57±13.18 | **<0.0001** |
|  | **B (P - Value) *** | -24.651 **(<0.0001)** | -9.971 **(<0.0001)** | -8.322 **(<0.0001)** | **Reference** |  |
|  | **B (P - Value) **** | -17.04 **(<0.0001)** | -16.52 **(<0.0001)** | -2.09 **(<0.0001)** | **Reference** |  |
|  | **B (P - Value) * & **** | -14.014 **(<0.0001) &** -11.89 **(<0.0001)** | | | **Reference** |  |

Group 1 = FCEM + CCM; Group 2 = FCEM; Group 3 = CCM; Group 4 = Control; Abbreviation; TLC: Total lung capacity; DLCO: Diffusing capacity of the lungs for carbon monoxide; FEV_1_: Forced expiratory volume in 1 second; FVC: Forced vital capacity; RV: Residual volume; *Generalized Estimation Equations (GEE), **Panel (Adjusted), *** ANOVA

**Table 5:** Standardized Direct, Indirect, and Total effects of independent variables on outcomes of Structural Equation Model (SEM)

| **Mediators** | | | | | | | | | | | | **Outcomes** | | | | | | **Effects** | |
| --- | --- | --- | --- | --- | --- | --- | --- | --- | --- | --- | --- | --- | --- | --- | --- | --- | --- | --- | --- |
| **BI** | | | **K10** | | | **FEV1/FVC** | **FEV1** | **FVC** | **RV** | **DLCO** | **TLC** | **6MWT** | | | **Walk Test** | | |  | |
| Total | Indirect | Direct | Total | Indirect | Direct | Direct | Direct | Direct | Direct | Direct | Direct | Total | Indirect | Direct | Total | Indirect | Direct |  |  |
| .150 | .114 | .036 | -.639 | .086 | -.726*** | .217*** | .378*** | .412*** | -.341*** | .398*** | .295** | .202 | -.155 | .357*** | .212 | -.167 | .380*** | **Pooled groups** | **Independent Variables** |
|  |  | .132 |  |  | -.019 | **Fitted model parameters:**  **Chi^2^+20.451 ,p-value=.085**  **Chi^2^/df=1.573**  **GFI=.993**  **AGFI=.965**  **NFI=.972**  **IFI=.990**  **TLI=954**  **CFI=989**  **RMSEA=.064** | | | | | |  | -.018 |  |  | -.018 |  | **TLC** |  |
|  |  | -.082 |  |  | .208* |  |  |  |  |  |  |  | .054 |  |  | .058 |  | **DLCO** |  |
|  |  | .017 |  |  | .034 |  |  |  |  |  |  |  | .005 |  |  | .006 |  | **RV** |  |
|  |  | .092 |  |  | -.008 |  |  |  |  |  |  |  | -.012 |  |  | -.012 |  | **FVC** |  |
|  |  | .116 |  |  | .007 |  |  |  |  |  |  |  | -.011 |  |  | -.011 |  | **FEV1** |  |
|  |  | .168 |  |  | .096 |  |  |  |  |  |  |  | .003 |  |  | .005 |  | **FEV1/FVC** |  |
|  | | | | | |  |  |  |  |  |  |  |  | .217* |  |  | .237** | **K10** |  |
|  |  |  |  |  |  |  |  |  |  |  |  |  |  | -.107 |  |  | -.105 | **BI** |  |

Abbreviations; TLC: Total lung capacity; DLCO: Diffusing capacity of the lungs for carbon monoxide; RV: Residual volume; FVC: Forced vital capacity; FEV_1_: Forced expiratory volume in 1 second; K10: Kessler psychological distress scale; BI: Barthel Index; Chi^2^: Chi-square; df: degrees of freedom; GFI: Goodness-of-fit index; AGFI: Adjusted GFI; NFI: Normed fit index; IFI: Incremental fit index; TLI: Tucker-Lewis index; CFI: Comparative fit index; RMSEA: Root mean square error of approximation

| **A**  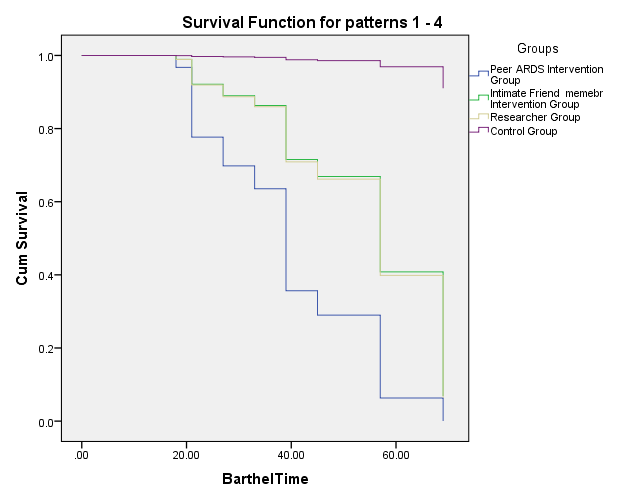 |
| --- |
| **B**  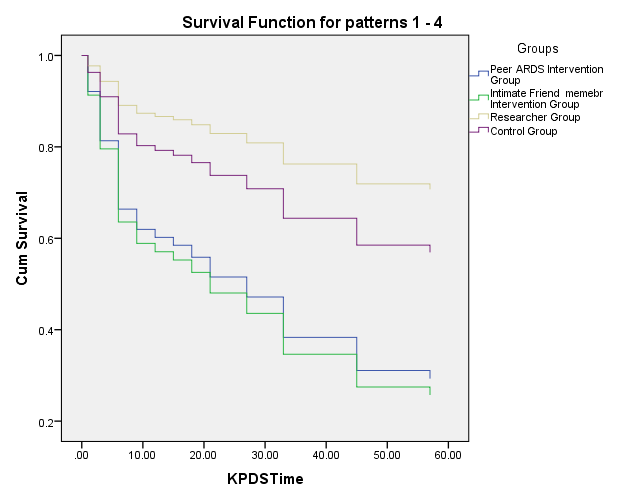 |

**Figure 2:** Multiple Cox survival analyses according to (A) Barthel index time and (B) Kessler Psychological Distress Scale (K10) time in four groups of study


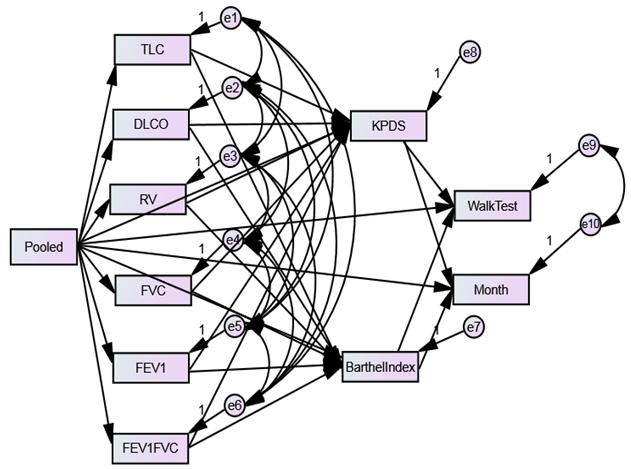


**Figure 3:**The Structural Education Modeling (SEM) for relationship between variables and waking index (WI) and Month as outcomes

**References**

1. Vahedian-azimi A, Alhani F, Goharimogaddam K, Madani S, Naderi A&Hajiesmaeili M. Effect of family - centered empowerment model on the quality of life in patients with myocardial infarction: A clinical trial study. *2 Journal of Nursing Education*. 4:8-22. <https://doi.org/> (2015).

2. Lins L&Carvalho FM. SF-36 total score as a single measure of health-related quality of life: Scoping review. *SAGE open medicine*. 4:2050312116671725. <https://doi.org/10.1177/2050312116671725> (2016).

3. Pfoh ER, et al. The SF-36 Offers a Strong Measure of Mental Health Symptoms in Survivors of Acute Respiratory Failure. A Tri-National Analysis. *Annals of the American Thoracic Society*. 13:1343-50. <https://doi.org/10.1513/AnnalsATS.201510-705OC> (2016).

4. Qu B, Guo HQ, Liu J, Zhang Y&Sun G. Reliability and validity testing of the SF-36 questionnaire for the evaluation of the quality of life of Chinese urban construction workers. *The Journal of international medical research*. 37:1184-90. <https://doi.org/10.1177/147323000903700425> (2009).

5. Motamed N, Ayatollahi AR, Zare N&Sadeghi-Hassanabadi A. Validity and reliability of the Persian translation of the SF-36 version 2 questionnaire. *Eastern Mediterranean health journal = La revue de sante de la Mediterranee orientale = al-Majallah al-sihhiyah li-sharq al-mutawassit*. 11:349-57. <https://doi.org/> (2005).

6. Fliege H, et al. The Perceived Stress Questionnaire (PSQ) reconsidered: validation and reference values from different clinical and healthy adult samples. *Psychosomatic medicine*. 67:78-88. <https://doi.org/10.1097/01.psy.0000151491.80178.78> (2005).

7. Levenstein S, et al. Development of the Perceived Stress Questionnaire: a new tool for psychosomatic research. *Journal of psychosomatic research*. 37:19-32. <https://doi.org/10.1016/0022-3999(93)90120-5> (1993).

8. Maroufizadeh S, Zareiyan A&Sigari N. Reliability and validity of Persian version of perceived stress scale (PSS-10) in adults with asthma. *Archives of Iranian medicine*. 17:361-5. <https://doi.org/> (2014).

9. Julian LJ. Measures of anxiety: State-Trait Anxiety Inventory (STAI), Beck Anxiety Inventory (BAI), and Hospital Anxiety and Depression Scale-Anxiety (HADS-A). *Arthritis care & research*. 63 Suppl 11:S467-72. <https://doi.org/10.1002/acr.20561> (2011).

10. Gustafson LW, et al. Validity and reliability of State-Trait Anxiety Inventory in Danish women aged 45 years and older with abnormal cervical screening results. *BMC medical research methodology*. 20:89. <https://doi.org/10.1186/s12874-020-00982-4> (2020).

11. Kancir CB&Korsgaard PK. Activities of daily living (Barthel Index) at discharge from the intensive care unit. *Critical Care*. 14:P439. <https://doi.org/10.1186/cc8671> (2010).

12. Sinoff G&Ore L. The Barthel activities of daily living index: self-reporting versus actual performance in the old-old (> or = 75 years). *Journal of the American Geriatrics Society*. 45:832-6. <https://doi.org/10.1111/j.1532-5415.1997.tb01510.x> (1997).

13. Anderson TM, Sunderland M, Andrews G, Titov N, Dear BF&Sachdev PS. The 10-item Kessler psychological distress scale (K10) as a screening instrument in older individuals. *The American journal of geriatric psychiatry : official journal of the American Association for Geriatric Psychiatry*. 21:596-606. <https://doi.org/10.1016/j.jagp.2013.01.009> (2013).

14. Andrews G&Slade T. Interpreting scores on the Kessler Psychological Distress Scale (K10). *Australian and New Zealand journal of public health*. 25:494-7. <https://doi.org/10.1111/j.1467-842x.2001.tb00310.x> (2001).

15. Hajebi A, et al. Adaptation and validation of short scales for assessment of psychological distress in Iran: The Persian K10 and K6. *International journal of methods in psychiatric research*. 27:e1726. <https://doi.org/10.1002/mpr.1726> (2018).

16. Graham BL, et al. Standardization of Spirometry 2019 Update. An Official American Thoracic Society and European Respiratory Society Technical Statement. *American journal of respiratory and critical care medicine*. 200:e70-e88. <https://doi.org/10.1164/rccm.201908-1590ST> (2019).

17. Macintyre N, et al. Standardisation of the single-breath determination of carbon monoxide uptake in the lung. *The European respiratory journal*. 26:720-35. <https://doi.org/10.1183/09031936.05.00034905> (2005).

18. Kline RB. Software programs for structural equation modeling: AMOS, EQS, and LISREL. *Psychoeducational Assessment*. 14:343-64. <https://doi.org/> (1998).
